# Co-Occurrence Relationship and Stochastic Processes Affect Sedimentary Archaeal and Bacterial Community Assembly in Estuarine–Coastal Margins

**DOI:** 10.3390/microorganisms10071339

**Published:** 2022-07-01

**Authors:** Yihong Yue, Yi Tang, Ling Cai, Zhihong Yang, Xueping Chen, Yurong Ouyang, Juanjuan Dai, Ming Yang

**Affiliations:** 1 School of Environmental and Chemical Engineering, Shanghai University, Shanghai 200444, China; yihongyue@shu.edu.cn (Y.Y.); tangyi@baiyi-tech.cn (Y.T.); yangzh22@shu.edu.cn (Z.Y.); xpchen@shu.edu.cn (X.C.); 2 Third Institute of Oceanography, Ministry of Natural Resources, Xiamen 361005, China; ouyangyurong@tio.org.cn (Y.O.); daijuanjuan@tio.org.cn (J.D.); 3 Observation and Research Station of Island and Coastal Ecosystems in the Western Taiwan Strait, Ministry of Natural Resources, Xiamen 361005, China

**Keywords:** microbial community, estuarine–coastal margins, methane metabolism, ammonia oxidation, co-occurrence, stochastic processes

## Abstract

Sedimentary microorganisms play crucial roles in maintaining the functional stability of aquatic ecosystems. However, their taxonomic composition and assembly processes are not well known in estuarine–coastal margins because of their complex environment. We investigated microbial communities, co-occurrence relationships, and underlying mechanisms in 33 surface sediment samples collected in the Jiulong River Estuary and the Taiwan Strait to reveal their composition dynamics. The abundance, diversity, and composition of microorganisms demonstrated obvious spatial variables. *Methanobacterium* and *Methanosarcina,* as well as Candidatus_*Nitrosopumilus* and *Nitrososphaeraceae* were the main methanogenic and ammonia-oxidizing archaea, with an average abundance of more than 5.91% and 4.27%, respectively. Along with a salinity gradient increase, the relative abundance of methanogenic archaea (from 42.9% to 16.6%) contrasted with the trend of ammonia-oxidizing archaea (from 6.04% to 18.7%). The number of methanogenic archaea gradually decreased with increasing geographic distance (*p* < 0.05), whereas ammonia-oxidizing archaea showed no significant change (*p* > 0.05). In co-occurrence patterns, closer inter-taxa connections were observed among archaea–archaea and bacteria–bacteria than in archaea–bacteria, which indicated that coexistence within the same kingdom was greater than interaction between different kingdoms in shaping the community structure along the salinity gradient. Furthermore, null model analyses of the microbial community showed that undominated was the most prominent process, explaining over 44.9% of community variation, followed by heterogeneous selection and dispersal limitation, which contributed to 27.7% and 16.3%, respectively. We demonstrated that stochasticity, rather than determinism, regulates community assembly. These results further highlight that intra-kingdom co-occurrence and stochastic processes shape the structure and assembly of microbial communities in estuarine–coastal margins.

## 1. Introduction

Estuarine–coastal margins are important channels connecting terrestrial and marine ecosystems, where rivers with substantial amounts of nutrients and pollutants are input and accumulated [1,2]. Complex and dynamic ecosystems with strong physicochemical gradients, enhanced biological activity, and intensive sedimentation and resuspension are formed in the estuarine–coastal margins [3]. This special aquatic environment shapes the unique sedimentary microbial community with functional diversity [4,5], which affects the ecological functions of methane metabolism [6], nitrification–denitrification [7], and organic matter degradation [8]. The impact of estuarine–coastal margins on microbial diversity and ecological processes is a critical issue in aquatic ecosystems; however, it has not been fully addressed.

Estuarine–coastal margins are affected by land–sea interactions, human activities, and ocean currents. Their complex physicochemical factors, rich nutrients, and diverse habitats have shaped a highly diverse microbial community. Substantial amounts of terrigenous organic matter and pollutants are continuously input and undergo rapid deposition–suspension–sedimentation, induced by hydrodynamic conditions such as tides. The high biodiversity and abundant substrates in the estuarine–coastal margins provide an ideal habitat for microorganisms, which enriches the microorganisms in the sediments. Research on microorganisms in estuarine–coastal margins has focused on the community structure and its ecological functions. Microorganisms form the core of the carbon [9], nitrogen [10,11], phosphorus [12], and sulfur [13] cycles, as well as other elements in sediments. They participate in biological processes, such as methane metabolism, ammonia oxidation [14], and organic matter decomposition, and play an indispensable role in the maintenance of ecosystem stability and environmental purification. As an important biological indicator for evaluating estuarine–coastal margins, it is crucial to comprehensively understand the changes in microbial community to elucidate their ecological roles in estuarine–coastal environments.

Estuarine–coastal margins, accounting for only 0.4% of the global marine area, are considered one of the most abundant biomass ecosystems. In recent years, attention has been paid to the microbial communities in estuarine–coastal margins worldwide. On a large scale, the distribution of microorganisms in estuarine–coastal margins is mainly affected by geographical features, including climate, latitude, and water depth [15]. On a small scale, it is mainly affected by the abiotic environmental factors of their habitat, such as salinity, temperature, dissolved oxygen, nutrients, pollutants, and other biotic factors associated with competition and predation [16,17]. When studying the macro-ecological pattern of marine bacteria on a global scale, Amend et al. [18] suggested that there is a significant positive correlation between sequence abundance and latitude, verifying the applicability of Rapoport’s rule in marine bacteria, while Liu et al. [19] highlighted the notion that the diversity of archaeal communities in low- and mid-latitude estuaries is significantly higher than that in high-latitude estuaries in over 20 estuaries. Furthermore, salinity mainly reflects the community composition in the sediments of different estuaries. Webster et al. [20] explored the sedimentary archaea in the Kern River Estuary and found that *Thaumarchaeota* and *Bathyarchaeota* preferentially dwell in high-salinity estuaries, whereas *Euryarchaeota* and *Bathyarchaeota* are phyla from predominantly low-salinity estuaries. Stochastic processes also play an important role in the ecological processes that regulate microbial distribution patterns [21]. Stochastic processes correspond to neutral theories and include ecological drift, probabilistic dispersal, and random speciation and extinction. Stochasticity exerts a stronger effect than determinism on the assembly of planktonic archaeal community in coastal wetlands [22], and bacterial [23] and archaeal [24] communities in coastal seawater. However, it is unclear whether stochastic processes dominate the assembly of microbial communities in the estuarine–coastal sediments. This knowledge is necessary for ensuring a comprehensive understanding of the microbial ecology in estuarine–coastal margins.

In this study, the Jiulong River Estuary and the Taiwan Strait, which are located in Southeastern China and are strongly disturbed by human activities, were selected in order to study the diversity and assembly of microbial communities in surface sediments. The objectives of this study were to: (1) analyze the abundance and diversity of archaeal and bacterial communities; (2) explore sedimentary archaea–bacteria co-occurrence patterns in estuarine–coastal margins; and (3) evaluate the respective contribution of deterministic and stochastic processes governing microbial community assembly.

## 2. Materials and Methods

### 2.1. Description of Study Area

The Jiulong River Estuary (24.38–24.50° N, 117.75–118.10° E), located in northwest Xiamen Bay, is the second largest river in Fujian Province, Southeast China. The catchment area of the Jiulong River Estuary is 1.47 × 10^4^ km^2^, the annual water temperature ranges from 13 °C to 32 °C, and the annual rainfall is 1400–1800 mm. The Jiulong River Estuary is a typical submerged tidal inlet estuary with small runoff, large tidal range, high salinity, and a hydrodynamic residence time of approximately 2 days [25]. An important water source for drinking, as well as agricultural and industrial activities in Southeast China, the Jiulong River Estuary flows southeast through Xiamen Bay and further into the southern Taiwan Strait.

The Taiwan Strait, located between Taiwan Island and mainland China, is an important route and transportation channel connecting the South China Sea and East China Sea. Due to strong ocean currents, coastal upwelling, and significant terrestrial input from the mainland and Taiwan Island, many terrestrial rivers flow into the Taiwan Strait from the mainland and Taiwan Island, transporting a large amount of nutrients and pollutants [26,27]. In summer, the Taiwan warm current and the Kuroshio Branch current transport seawater from the South China Sea to this region. In contrast, during winter, due to the East Asian monsoon, the Zhejiang–Fujian Coastal Current is the strongest [28].

### 2.2. Samples Collection

**Figure 1:**
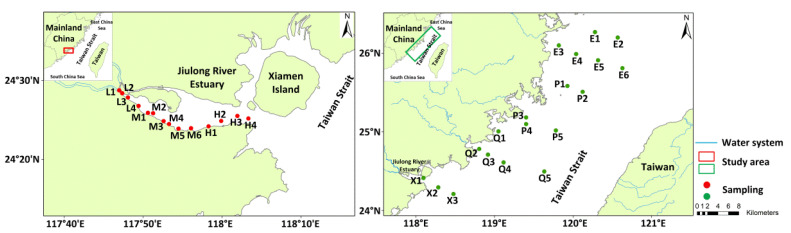
Location and distribution of sampling sites in the Jiulong River Estuary and the Taiwan Strait in China.

As shown in Figure 1, surface sediments (top 0–5 cm) were collected at 33 sampling sites located in the Jiulong River Estuary and Taiwan Strait in January 2019. According to geomorphologic and salinity gradients, 14 stations were divided into three parts along the South Bank of the Jiulong River Estuary, including the low-salinity area (L), which is characterized by a narrow channel and freshwater outflow; the medium-salinity area (M), which is an expanse of water bodies with brackish water; and a high-salinity area (H), which is near the mouth of the estuary and are mainly mixed with seawater [6]. According to the geographical distance from the estuary, 19 stations (X, Q, P, and E) were arranged in the Taiwan Strait. The sediment samples were divided into two halves: one half was stored in sample bags at 4 °C to measure physicochemical parameters, and the other half was stored at −20 °C for microbial analysis. Three replicates were collected for each sediment sample.

### 2.3. Physicochemical Analysis

Water temperature (Temp), dissolved oxygen (DO), salinity, pH, total dissolved solids (TDS) of bottom water, chlorophyll a (Chla), and a blue green algae-phycoerythrin (BGA-PE) of surface water were measured in situ with a YSI water quality analyzer (Weiser Pro 2030, USA). The sediment samples were immediately freeze-dried to detect total nitrogen (TN), total organic carbon (TOC), and ammonium nitrogen (NH_3_-N). Dried sediment (1 g) was added to 5 mL hydrochloric acid at a concentration of 2 mol/L for 24 h to remove inorganic carbon. Then, centrifugation was carried out to accelerate precipitation and remove residual acid; the sample was washed until a neutral pH was attained. After re-freeze-drying, sediment TN and TOC were analyzed using an elemental analyzer (Flash 2000HT, Thermo Fisher Scientific, Waltham, MA, USA) [29]. In the presence of an alkaline medium and sodium nitroprusside, ammonium in the sediment reacts with sodium salicylate and sodium dichloroisocyanurate to form a colored complex. The NH_3_-N content of the sediments was calculated by measuring the absorbance at 660 nm. All measurements were performed in triplicate. Detailed information regarding the environmental parameters is listed in Table S1.

### 2.4. DNA Extraction, Polymerase Chain Reaction (PCR), and Sequencing

Total DNA was extracted from a 0.25 g wet sediment sample using a FastDNA SPIN Kit for soil (MP Biomedicals, USA), according to the manufacturer’s protocols. The quality and quantity of the extracted DNA were determined using the Qubit 3.0 fluorometer (Thermo Fisher Scientific, Waltham, MA, USA). The primer sets 524F10extF (5′-TGYCAGCCGCCGCGGTAA-3′)/Arch958RmodR (5′-YCCGGCGTTGAVTCCAATT-3′) [30] and 338F (5′-ACTCCTACGGGAGGCAGCAG-3′)/806R (5′-GGACTACHVGGGTWTCTAAT-3′) [31] were used to amplify the V4–V5 region of archaea and V3–V4 region of bacteria, respectively. Triplicate amplifications were performed in a 25 μL reaction system containing a unit of *TransStart*^®^  *FastPfu* DNA Polymerase (TransGen, Beijing, China), 2.5 mM of dNTPs, 5 μL of 5× buffer, 50 mM of Mg^2+^, 0.1 μM of each primer, and 1 ng of the DNA template. PCR products were purified using Agencourt AMPure XP beads (Beckman, CA, USA). The library of three duplicate samples was mixed as a DNA library of one sampling site, and the DNA library of each sampling site was sequenced twice. Sequencing was performed using the Illumina MiSeq PE300 platform (Illumina, San Diego, CA, USA).

### 2.5. Quantitative PCR (qPCR)

Universal primers A364Af/A934bR [32] and 341f/534r [33] were used to amplify archaeal and bacterial 16S rRNA genes, respectively. The PCR fragment was ligated into the pMD18-T vector to form plasmid standards. qPCR was performed in triplicate amplifications using the Bio-Rad CFX384 Real-Time PCR Detection System (Bio-Rad Laboratories Inc., Hercules, CA, USA) and TB Green Fast qPCR Mix (TaKaRa, Tokyo, Japan). The qPCR amplification conditions included an initial denaturation at 95 °C for 10 min, 40 cycles of denaturation at 95 °C for 15 s, annealing at 60 °C for 1 min, followed by 95 °C for 5 s, and 65 °C for 5 s of melting curve. Standard curves were generated with 10 fold serial dilution of plasmids, and the abundance of microbial 16S rRNA gene was calculated based on the cycle threshold (CT) value and plasmid standard curve. The average amplification efficiencies of the archaea and bacteria were 91.9% and 100.5%, respectively.

### 2.6. Sequence Denoising, Operational Taxonomic Units (OTU) Clustering, and Diversity Analysis

The raw data of FASTQ obtained from sequencing were processed in the execution environment of QIIME2 (http://qiime.org/scripts/assign_taxonomy.html (accessed on 9 April 2021)). After removing the sequenced primers and adapter sequences, the paired reads were merged into long sequences, filtered, and denoised according to sequence quality. High-quality sequences were compared with the SILVA database (Release138, http://www.arb-silva.de (accessed on 2 March 2022)) to discard eukaryotic, bacterial, and archaeal sequences. The screened sequences were then clustered into operational taxonomic units (OTUs) based on a 99% similarity standard. For sequencing depth, each sample was rarefied to 30,000, which was the lowest sequence number across all samples. Using the Mothur software (version 1.30.2, https://www.mothur.org/wiki/Download_mothur (accessed on 2 March 2022)), the sample alpha diversity index, including Good’s coverage, richness indices, and diversity indices, were determined. Principal co-ordinates analysis (PCoA) was performed using the unweighted UniFrac distance metric to compare the similarities and differences in the microbial community of environmental samples with R software (version 3.3.1).

### 2.7. Variation Partitioning Analysis (VPA), Null Model and Network Analysis

Variation partitioning analysis (VPA) was applied to evaluate the proportion of environmental factors and their combined effect by using the “vegan” libraries in R. Null model was performed to determine the contribution of determinism and stochasticity to community assembly. The ecological processes were calculated using a standardized estimate of beta nearest taxon index (βNTI) [34]. |βNTI| > 2 and |βNTI| < 2 indicate determinism- or stochasticity- dominated community composition, respectively [35]. A co-occurrence network was constructed using the “WGCNA” libraries in R and visualized with the “Gephi 0.9.2” software (http://gephi.org (accessed on 2 March 2022)). Network analysis illustrated the co-occurrence patterns of the archaeal and bacterial taxa [36,37]. To reduce complexity, only genera with the top 30 of the archaeal and bacterial relative abundance across all samples were retained, respectively. Pearson correlation and significance analyses were used to examine the potential links among different samples according to the r and *p* values using SPSS 20.0 (IBM, Armonk, NY, USA). All statistical analyses were performed using R software (version 3.3.1).

## 3. Results

### 3.1. Biogeochemical Parameters in the Sediments

The environmental parameters of the sediment samples in the Jiulong River Estuary and the Taiwan Strait were measured (Table S1). Based on salinity, 14 sampling points of the Jiulong River Estuary were divided into low-salinity (L, salinity of 5.63 ± 3.02‰), medium-salinity (M, 14.6 ± 1.76‰) and high-salinity (H, 24.1 ± 2.51‰) areas. According to the direction of the ocean current from the north to the south, 19 samples were collected at four stations, E, Q, P, and X in the Taiwan Strait (Table 1). There were obvious spatial differences in the physicochemical properties of the Jiulong River Estuary sediments along the salinity gradient (*p* < 0.05). Water DO and TDS ranged from 6.45 to 8.08 mg/L and 6404 to 24,639 mg/L, respectively, with higher values in H and M than that in L for most of the sampling sites (*p* < 0.05). The Chla, BGA-PE, and NH_3_-N contents were the highest in L, followed by M, and the lowest in H. The TN and TOC contents in the sediments ranged from 0.0733 to 0.133 % and 0.855 to 1.19 %, and mostly had higher values in L than that in H and M (*p* < 0.05). There were no significant differences in the environmental parameters of the Taiwan Strait at the sampling points (*p* > 0.05). The water depth, temperature, salinity, and TDS in the Taiwan Strait were significantly higher than those in the Jiulong River Estuary, whereas Chla, BGA-PE, NH_3_-N and TOC in the Taiwan Strait were significantly lower than those in the Jiulong River Estuary (*p* < 0.05). These results indicated that the Jiulong River Estuary had significant spatial differences as compared to the Taiwan Strait.

### 3.2. Abundance and Diversity of Bacterial and Archaeal Community

**Figure 2:**
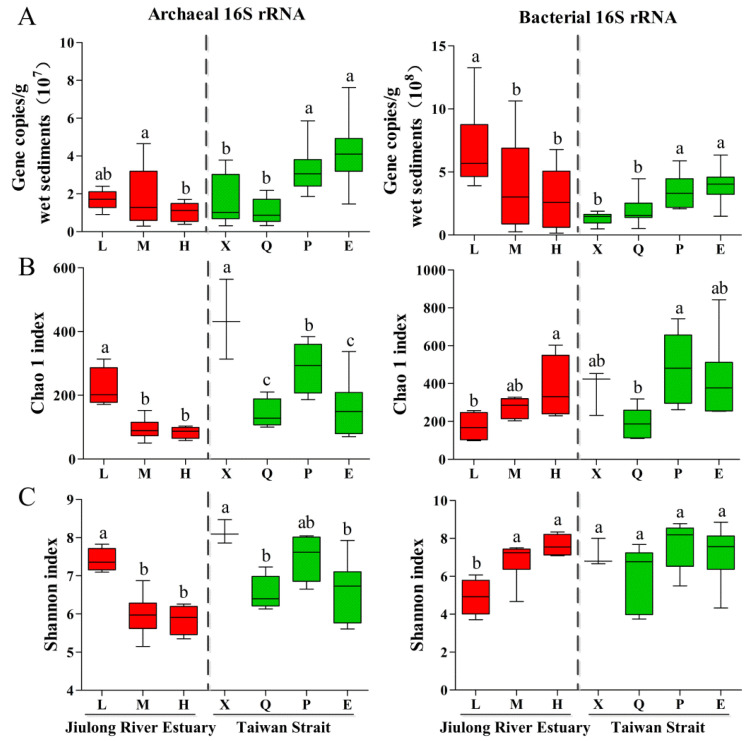
The microbial abundance and diversity in sediments of the Jiulong River Estuary and the Taiwan Strait. 16S rRNA gene copies derived from qPCR (**A**) and indices of alpha diversity shown as Chao 1 (**B**) and Shannon (**C**). Numbers in a rank with different letters indicate a significant difference (Duncan’s Test, *p* < 0.05).

The absolute abundance of archaeal 16S rRNA genes in the sediments of the Jiulong River Estuary and the Taiwan Strait varied from 3.0 × 10^6^ to 4.7 × 10^7^ copies/g of wet sediment, while bacterial 16S rRNA gene copies ranged from 1.5 × 10^7^ to 1.3 × 10^9^ copies/g of wet sediment (Figure 2). Bacterial abundance was significantly higher than archaeal abundance (*p* < 0.05), with copy numbers one to three orders of magnitude higher. The archaeal copy number was the lowest in H of the Jiulong River Estuary, which was slightly lower than that in L and M (*p* < 0.05) (Figure 2A). However, the highest number of bacterial copies was observed in L of the Jiulong River Estuary, which was significantly higher than those in M and H (*p* < 0.05). Microbial abundance increased significantly with geographical distance from the estuary (Figure 2B). Most sediment samples in the Taiwan Strait had significantly higher numbers of archaeal copies than those in the Jiulong River Estuary (*p* < 0.05), whereas the bacterial abundance in the Jiulong River Estuary was significantly higher than that in the Taiwan Strait (*p* < 0.05). Overall, there was an obvious spatial variation in the microbial abundance in both the Jiulong River Estuary and the Taiwan Strait.

A total of 5970 archaeal OTUs and 10,049 bacterial OTUs in the sediment of the Jiulong River Estuary and the Taiwan Strait were classified with 99% of them identified after filtering the high-throughput sequencing. Alpha diversity indices are shown in Table S2. The Chao1 indices of the sediment in the archaeal and bacterial communities were 50 to 564 and 99 to 843, respectively. The Shannon indices of the sediment in archaeal and bacterial communities were 5.15 to 8.47 and 3.71 to 8.85, respectively. The Shannon index of L was the highest in archaea but the lowest in bacteria, and those of the M and H of the Jiulong River Estuary were not significantly different (Figure 2C). The Shannon index trends of archaea and bacteria in the Jiulong River Estuary were the opposite. Sedimentary microbes showed contrasting trends between the Shannon index and abundance in both the Jiulong River Estuary (except for archaea) and the Taiwan Strait (*p* < 0.05). In addition, the microbial Shannon index in the Taiwan Strait was significantly higher than that in the Jiulong River Estuary (*p* < 0.05). Similarly, at the OTU level, principal co-ordinates analysis (PCoA) explained 55.2% and 65.7% variances in archaeal and bacterial communities, respectively, which showed the obvious spatial distribution of microbial compositions (*p* = 0.001). Therefore, the Jiulong River Estuary and the Taiwan Strait showed significant spatial differences in microbial alpha and beta diversities (Figure S1).

### 3.3. Taxonomy and Composition of Bacterial and Archaeal Community

**Figure 3:**
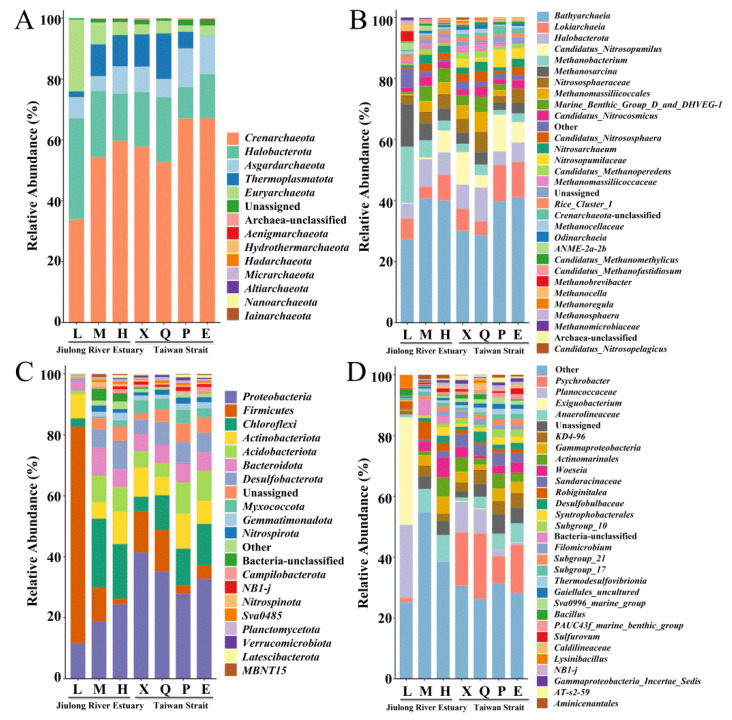
The composition of dominant microbial phyla (**A**,**C**) and genera (**B**,**D**) in sediments of the Jiulong River Estuary and the Taiwan Strait.

The composition of the microbial communities in the sediment samples was analyzed using 16S rRNA gene high-throughput sequencing (Figure 3). For the archaea communities, the dominant phylum was *Crenarchaeota,* with an abundance ranging from 29.5% to 82.1%, followed by the next phylum, *Halobacterota*, with the relative abundance ranging from 2.63% to 36.8% (Figure 3A). At the genus level, *Bathyarchaeia* dominated all the samples, with relative abundances ranging from 15.1% to 60.3% (Figure 3B). Among the top 30 archaea genera, there were 13 methanogenic archaea and 7 ammonia-oxidizing archaea. *Methanobacterium* and *Methanosarcina* were the main methanogenic archaea, with an average abundance of more than 5.91%. With an increase in salinity in the Jiulong River Estuary, the abundance of methanogenic archaea gradually decreased. With an increase in the geographical distance from the estuary in the Taiwan Strait, the abundance of methanogenic archaea gradually decreased, except at site X. Candidatus_*Nitrosopumilus* and *Nitrososphaeraceae* were the main ammonia-oxidizing archaea, with an average abundance of more than 4.27%. Contrary to the abundance of methanogenic archaea, the abundance of ammonia-oxidizing archaea gradually increased with the increase in salinity of the Jiulong River Estuary. At the four stations in the Taiwan Strait, although there were obvious spatial differences among different genera of ammonia-oxidizing archaea, their total content was 22.2–25.6% with no significant differences (*p* > 0.05). Therefore, with changes in salinity, the change in trends of methanogenic archaea and ammonia-oxidizing archaea were contrasting. With an increase in geographical distance from the estuary, the abundance of methanogenic archaea gradually decreased, while that of ammonia-oxidizing archaea showed no significant change.

*Firmicutes* was the most predominant bacterial phylum in L of the Jiulong River Estuary, with a relative abundance of more than 80%, while the dominant phylum in the other sites was *Proteobacteria* with an abundance ranging from 8.59% to 69.1% (Figure 3C). In the Jiulong River Estuary, *Exiguobacterium* and *Planococcaceae* were the dominant genera in L with an average abundance of 35.4% and 24.0%, respectively, which were lower than 0.0668% in both M and H. *Anaerolineaceae*_uncultured was the dominant genus in M and H, with an average abundance of 7.72% and 8.70%, respectively (Figure 3D). With an increase in salinity, the abundances of *Actinomarinales*_uncultured, *Woeseia*, *Sandaracinaceae_*uncultured, *Subgroup_10*, *Subgroup_21*, *NB1-j*, *Caldilineaceae*, *Syntrophobacterales*_uncultured, *Sulfurovum*, *PAUC43f_marine_benthic_group*, *Gaiellales*_uncultured, and *Desulfobulbaceae*_uncultured gradually increased. *Psychrobacter* was the dominant genus in the Taiwan Strait, with an average abundance of 16.0%, whose abundance in the Jiulong River Estuary was less than 1.52%. With an increase in geographical distance from the estuary, the abundance of *Planococcaceae* decreased gradually, while that of *Anaerolineaceae*_uncultured, *Syntrophobacterales*_uncultured, and *Subgroup_21* gradually increased. Therefore, there were obvious spatial differences in the dominant bacterial phyla and genera between the Jiulong River Estuary and the Taiwan Strait.

### 3.4. Archaeal and Bacterial Co-Occurrence Network Analysis

**Figure 4:**
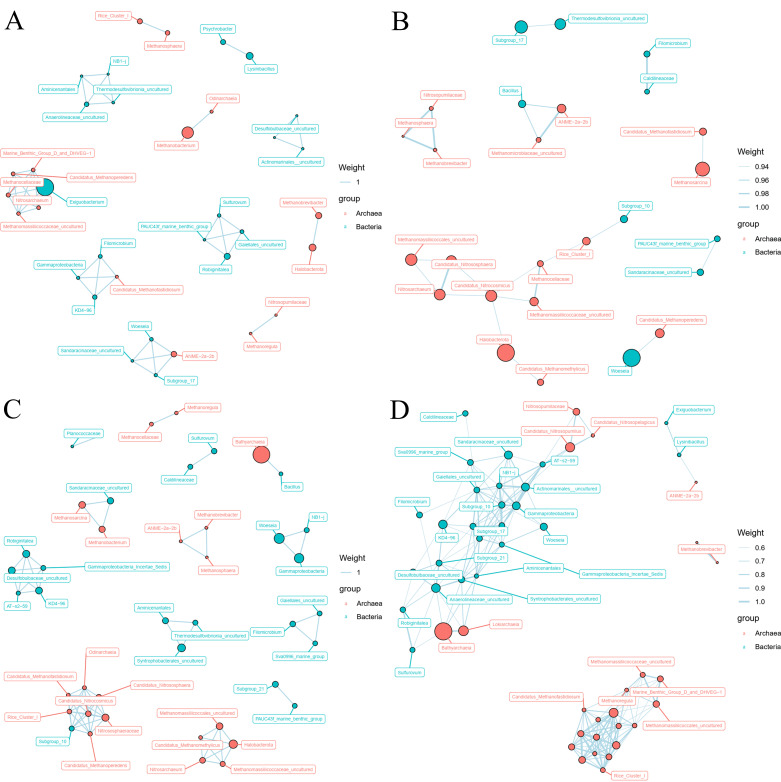
Co-occurrence networks of the sedimentary archaea and bacteria in low-salinity area (**A**), medium-salinity area (**B**), high-salinity area (**C**) of the Jiulong River Estuary and in the Taiwan Strait (**D**) based on pairwise Spearman’s correlations. The size of each node is proportional to the number of connections.

To identify the correlation between archaea and bacteria in the sediment samples, a co-occurrence network was constructed using the dominant prokaryotic genera (Figure 4). Microbial nodes were the top 30 genera in the community-relative abundance. The microbial community in the network was organized into archaea–archaea, bacteria–bacteria and archaea–bacteria. In the L of the Jiulong River Estuary, *Methanobacterium* with the highest archaeal relative abundance interacted with *Odinarchaeia*, while *Esiguobacterium*, with the highest bacterial-relative abundance, interacted with multiple methanogenic and ammonia-oxidizing archaea (Figure 4A). In the M of the Jiulong River Estuary, the interaction between multiple methanogenic and ammonia-oxidizing archaea were observed. *Woeseia* and *Bacillus* were related to Candidatus_*Methanoperedens* and *Methanomicrobiaceae*_*uncultured*, respectively (Figure 4B). In the H of the Jiulong River Estuary, *Bathyarchaea*, with the highest archaeal abundance, interacted with *Bacilus*, while *subgroups_10* interacted with multiple methanogenic and ammonia-oxidizing archaea (Figure 4C). In the Taiwan Strait, the interactions were mainly in the form of archaea–archaea and bacteria–bacteria (Figure 4D). Therefore, with the increase in salinity, both archaea and bacteria interacted more with their own genera than with each other. In addition, the degree and closeness centrality values of node-level topological features were significantly higher in the Taiwan Strait network than those in the Jiulong River Estuary network. These results indicated that microbial co-occurrence was more connected in the Taiwan Strait than in the Jiulong River Estuary.

### 3.5. Ecological Processes Governing Community Structure

**Figure 5:**
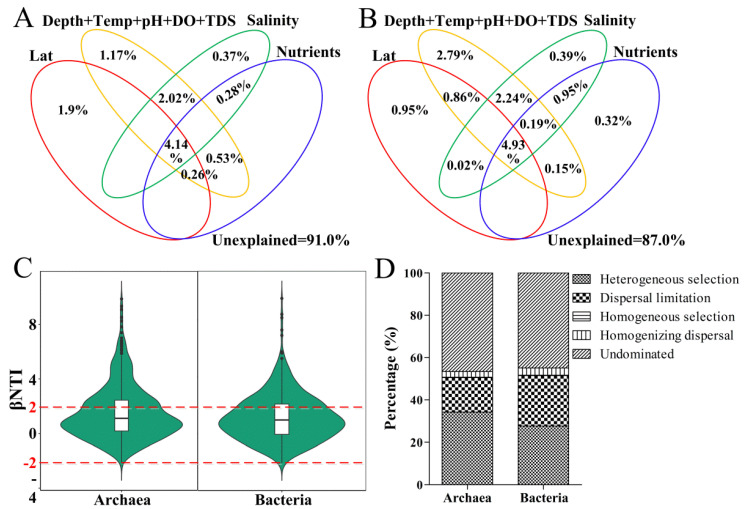
Variation partitioning analysis (VPA) (**A**,**B**) and null model analysis (**C**,**D**) revealing the assembly processes of the archaeal and bacterial community.

The relationship between microbial community dissimilarity and geographic distance illustrated a clear distance–decay pattern (*p* < 0.05) in both the Jiulong River Estuary and the Taiwan Strait, demonstrating increased community dissimilarity with increasing geographic distance, except for the bacteria in the Taiwan Strait (Figure S2). Variation partitioning analysis (VPA) was used to explore the relationship between the microbial community composition and environmental factors (Figure 5A,B). For the archaeal community, the effects of the salinity gradient, the pure environmental variables (e.g., depth, temperature, pH, DO, TDS) and the pure spatial processes (latitude, longitude) explained mean 0.37%, 1.17% and 1.9% of the community variations, respectively. For the bacterial community, the effects of the salinity gradient, the pure environmental variables, the pure spatial processes and nutrients (NH_3_-N, TN, TOC) explained mean 0.39%, 2.79%, 0.95% and 0.32% of the community variations, respectively. The overlapping area which represented the shared effects of two or more factors explained the community variations. Consequently, the unexplained archaeal and bacterial compositions accounted for 91.0% and 87.0%, respectively, which suggested that they may be stochastic processes rather than environmental factors shaping the microbial community composition.

The null model was used to verify the VPA results (Figure 5C). Archaea and bacteria βNTI scores ranged from −2 to +2, indicating that stochastic processes were greater than deterministic processes in microbial community assembly. For stochastic processes, undominated was prominent in the archaeal (46.6%) and bacterial (44.9%) community assembly, followed by a dispersal limitation of 16.3% and 23.9%, respectively (Figure 5D). βNTI was grouped according to the salinity and geographic distance (Figure S3). Bacterial βNTI increased with the increase in salinity in the Jiulong River Estuary, which decreased gradually with increasing geographic distance except for site X in the Taiwan Strait, indicating a decrease in the influence of stochastic processes on bacterial community assembly with an increase in salinity gradient, and an increase in stochastic processes with the increase in geographical distance. However, this phenomenon was not observed in archaeal communities.

## 4. Discussion

The estuarine–coastal margin is a typical ecosystem that is different from terrestrial freshwater and the ocean, and its surface sediments are the most active areas of microbial geochemical processes [21,38] with high primary productivity and biogeochemical cycles [13,39]. As a land–ocean transition zone, estuarine–coastal margins have obvious salinity gradients and abundant nutrients, thus presenting a unique microbial composition that differs from that of freshwater and oceans.

### 4.1. Characteristics of Microbial Community in the Sediments of Estuarine–Coastal Margins

Methanogenic archaea and ammonia-oxidizing archaea are ubiquitous in diverse ecosystems and play pivotal roles in the global carbon and nitrogen cycling. In this study, the archaeal sediment community was dominated by *Crenarchaeota* and fewer methanogens in the mid- or high-salinity regions of the estuary, whereas methanogenic *Halobacterota* [40] and *Euryarchaeota* were dominant in the low-salinity region of the estuary. Estuarine–coastal margins, despite their small surface area, emit approximately 75% of global oceanic CH_4_ emissions [41]. Some studies have found that the CH_4_ concentration in the Jiulong River Estuary is significantly negatively correlated with salinity [6]. In this study, the relative abundance of methanogenic archaea decreased with an increase in salinity in the Jiulong River Estuary, which was consistent with the methane concentration results. Estuaries exhibit strong gradients along their waterways, with organic matter and nitrogen concentrations decreasing with an increase in distance from the estuary head, whereas chloride and sulfate concentrations increase with a decrease in distance from the estuary mouth [20,42]. Most studies have shown that salt increases the number of electron acceptors in sediments. Although higher Cl^−^ was toxic for methanogens and reduced their abundance [43], it competed with methanogens for substrate sources, and thus inhibited the activity of methanogens, thereby reducing the CH_4_ emission flux. In addition, the concentration of SO_4_^2−^ available to sulfate-reducing bacteria increased with increasing salinity, which promoted anaerobic methane oxidation and inhibited the activity of methanogens. The affinity of sulfate-reducing bacteria for substrates, such as acetate and hydrogen, was higher than that of methanogens [44,45]. The abundance of *Desulfobacterota* and *Syntrophobacterales* detected in this study also confirmed that sulfate-reducing bacteria increased with increasing salinity. In the Taiwan Strait, the abundance of methanogenic archaea gradually increased with increasing geographical distance from the estuary, which may be due to human activities, such as sewage discharge and agricultural runoff. These activities enriched the organic matter in the sediments and thus, oxygen was consumed in the organic matter degradation process in the upstream. Furthermore, methanogenesis can only occur under strictly anaerobic conditions; ocean current activity increases the water level, which in turn enhances methanogenesis [46]. Estuarine methanogenesis is thus greatly influenced by salinity intrusion and river organic supply [47].

Archaea are the major ammonia oxidizers in brackish sediments and important contributors to estuarine nitrogen cycles [48]. Interestingly, in this study, the relative abundance of ammonia-oxidizing archaea in the Jiulong River Estuary increased with increasing salinity, which contrasted with the trend observed for methanogenic archaea. A previous study quantified the abundance of ammonia-oxidizing archaea (AOA) genes in the sediments of the Jiulong River Estuary, and observed that this abundance increased with increasing salinity [49]. With the salinity gradient and estuarine tidal action, the degradation of organic matter accelerated, and the oxygen supply in the sediment was insufficient. Under the action of microorganisms, NO_3_^-^ was reduced to NH_4_^+^, resulting in an increase in ammonium nitrogen content.

In terms of bacterial community composition, *Firmicutes* and its corresponding genera dominated the low-salt area of the Jiulong River Estuary, while *Proteobacteria* was the dominant bacterial phylum at the other sampling sites (Figure 3C,D). A previous study in estuaries also observed that *Firmicutes*, *Actinomyces*, and *Bacteroidetes* were highly abundant in low-salinity regions, whereas *Proteobacteria* and their corresponding alpha-*Proteobacteria* and gamma-*Proteobacteria* increased and predominated in abundance toward the coastal ocean [16]. Recently, research focused on salinity-related metabolic pathways of estuarine bacteria, such as bacterial respiration, glycolysis, osmoregulation, and metal transport, has revealed genomic differences between freshwater and marine bacteria through metagenomic studies [50,51]. Glycolysis is an important pathway for bacteria to produce energy, and the Embden–Meyerhof (EMP) pathway is common in *Actinomyces*, *Bacteroidetes*, and *Firmicutes*, whereas the Entner–Doudoroff (ED) pathway is prevalent in alpha-*Proteobacteria* and gamma-*Proteobacteria* [52]. As the key enzyme of the EMP pathway, 6-phosphofructokinase decreased with increasing salinity, while the key enzyme of the ED pathway, 2-keto-3-deoxy-6-phosphogluconate aldolase, was highly expressed in coastal oceans [16], which in turn shaped the different glycolysis pathways and the corresponding dominant bacterial communities across the salinity gradient. To this end, bacterial community studies in estuarine–coastal margins directly reflect the functional activity of microbial communities across salinity gradients.

### 4.2. Mechanisms Underlying the Microbial Assembly in the Sediments of Estuarine–Coastal Margins

Across estuarine–coastal margin transects, freshwater flows and tidal cycles fluctuate periodically under local conditions [48], leading to the redistribution of nutrients [53] and the formation of salinity gradients [54]. Salinity has been reported to be a major factor affecting the bacterial [55] and archaeal community composition [56]. However, in this study, despite the taxonomic differences in the communities under the salinity gradient, the VPA and null models found that stochastic processes shape archaeal and bacterial communities in the estuarine–coastal margins (Figure 5). The similarity between archaeal and bacterial communities in the Jiulong River Estuary decreased with increasing geographic distance, showing clear spatial differences (Figure S1). Sediment was a more heterogeneous environment than water, which was more evenly mixed during hydrodynamic movements [57,58]. The strong spatial heterogeneity of microbial communities might be due to higher stability within sediments, which were less affected by tidal mixing [59,60], while increasing geographic distance would weaken the ability of microorganisms to disperse and drift [61].

Numerous previous studies have explored the co-occurrence of archaea and bacteria in the same habitat and their functional interdependencies [22,62]. However, network analysis found that the coexistence of archaea–archaea and bacteria–bacteria increased with the salinity gradient in estuarine–coastal margins, rather than archaea–bacteria coexistence (Figure 4). This may be due to the unique environment of the estuarine–coastal margins, characterized by large amounts of organic and inorganic matter deposition. Microbes use rich nutrients in their habitat to fulfill their growth requirements rather than relying on decomposing substrates and obligate symbiosis between different kingdoms to ensure coexistence. Salinity is considered a stress factor for microbial communities [63]. The impacts of salinity on archaeal communities across estuarine–coastal margins are focused on the cycling of carbon, nitrogen, sulfur, and phosphorus [49], while bacterial communities were reflected in metabolic pathways, such as respiratory activity, membrane polarization and integrity, and glucose catabolism [50,64]. Therefore, the microbial communities that perform specific functions are interdependent. In addition, compared with the Jiulong River Estuary, the closer interactions among communities in the Taiwan Strait may be attributable to its fixed salinity, circulating nutrients, and stable sediment environment, which were more conducive to maintaining microbial ecosystem structure and functions.

## 5. Conclusions

Estuarine–coastal margins are complex and dynamic ecosystems influenced by periodic fluctuations of freshwater flow and tidal cycles. The abundance, diversity, and composition of sedimentary microorganisms in the Jiulong River Estuary and the Taiwan Strait showed clear spatial variation. The composition of sedimentary microorganisms of the former varied with the salinity gradient, whereas the latter was affected by geographical distance from the estuary. Co-occurrence of both archaea–archaea and bacteria–bacteria, rather than that of archaea–bacteria, played a key role in their community structure. Moreover, despite the taxonomic differences in the communities based on the salinity gradient, stochastic processes played a significant role in shaping microbial community assembly in estuarine–coastal margins. This may be because the sediments were less affected by tidal mixing, which weakened the dispersion and drift abilities of microorganisms. Thus, this study provides important implications for complex microbial dynamic patterns and inter-taxa interactions in estuarine–coastal margins.

## Figures and Tables

**Table 1:** The physicochemical characteristics of sediments in the Jiulong River Estuary and the Taiwan Strait *.

Area	Classifi-Cation	Depth (m)	Temp (°C)	Sal (‰)	pH	Chla (μg/L)	DO (mg/L)	BGA-PE (μg/L)	TDS (mg/L)	NH_3_-N (mg/kg)	TN (%)	TOC (%)
Jiulong River Estuary	L	8.93 ± 1.58 ^c^	17.7 ± 0.319 ^b^	5.63 ± 3.02 ^d^	7.72 ± 0.0572 ^c^	3.79 ± 0.541 ^a^	6.45 ± 0.379 ^c^	9 ± 2.03 ^a^	6404 ± 3225 ^d^	108 ± 9.6 ^a^	0.133 ± 0.0126 ^a^	1.19 ± 0.0755 ^a^
	M	6.95 ± 1.49 ^c^	17.2 ± 0.193 ^b^	14.6 ± 1.76 ^c^	7.8 ± 0.0921 ^c^	2.85 ± 0.273 ^b^	7.47 ± 0.25 ^b^	7.47 ± 0.669 ^b^	15,575 ± 1715 ^cd^	52.5 ± 37.3 ^b^	0.0733 ± 0.0484 ^b^	0.855 ± 0.383 ^ab^
	H	9.3 ± 1.46 ^c^	16.9 ± 0.164 ^b^	24.1 ± 2.51 ^b^	8.03 ± 0.0265 ^b^	2.37 ± 1.06 ^bc^	8.08 ± 0.0606 ^a^	5.74 ± 0.256 ^c^	24,639 ± 2332 ^bc^	32 ± 21.1 ^bc^	0.083 ± 0.0208 ^b^	0.88 ± 0.0321 ^b^
Taiwan Strait	X	26.5 ± 15.21 ^bc^	21.42 ± 0.68 ^a^	31.11 ± 1.1 ^a^	NA	NA	NA	NA	NA	21.62 ± 7.99 ^c^	0.06 ± 0.05 ^b^	0.51 ± 0.32 ^b^
	Q	37.42 ± 15.02 ^ab^	22.4 ± 1.54 ^a^	32.38 ± 1.53 ^a^	8.13 ± 0.05 ^a^	1.67 ± 0.32 ^c^	7.36 ± 0.11 ^b^	4.21 ± 0.66 ^d^	31,274.5 ± 1050.75 ^ab^	33.02 ± 14.05 ^bc^	0.06 ± 0.04 ^b^	0.47 ± 0.31 ^b^
	P	45.6 ± 19.76 ^ab^	21.43 ± 1.66 ^a^	31.95 ± 1.9 ^a^	NA	NA	NA	NA	NA	20.28 ± 5.98 ^c^	0.08 ± 0.03 ^ab^	0.6 ± 0.25 ^b^
	E	52.53 ± 18.23 ^a^	22.31 ± 1.2 ^a^	32.16 ± 1.88 ^a^	8.13 ± 0.04 ^a^	1.54 ± 0.3 ^c^	7.25 ± 0.34 ^b^	4.28 ± 0.59 ^d^	38,532.25 ± 14,846.99 ^a^	19.9 ± 4.17 ^c^	0.11 ± 0.03 ^ab^	0.76 ± 0.16 ^ab^

* Numbers in a rank with different letters indicate a significant difference (Duncan’s Test, *p* < 0.05).

## Data Availability

Raw sequence data obtained from Illumina MiSeq in this study has been deposited in the public NCBI database (http://www.ncbi.nlm.nih.gov/ (accessed on 9 April 2022)) under the accession numbers PRJNA847698 (archaea) and PRJNA847764 (bacteria).

## Supplementary Materials

The following supporting information can be downloaded at: https://www.mdpi.com/article/10.3390/microorganisms10071339/s1, Table S1: The environmental variables of sediments at sampling points in the Jiulong River Estuary and the Taiwan Strait; Table S2: Sequences number and alpha diversity indices of sediments in the Jiulong River Estuary and the Taiwan Strait; Figure S1: Principal co-ordinate analysis (PCoA) of the archaeal (A) and bacterial (B) communities in the Jiulong River Estuary and the Taiwan Strait, based on the unweighted UniFrac distance metric. Different circles indicate sediments samples were divided into different cluster patterns; Figure S2: Distance–decay patterns of the archaeal (A) and bacterial (B) community and geographical distance in the Jiulong River Estuary and the Taiwan Strait, based on Bray–Curtis dissimilarity. The black line indicates the fit between geographical distance and Bray–Curtis dissimilarity; Figure S3: Null model analysis revealing the assembly processes of archaeal (A) and bacterial (B) community in the Jiulong River Estuary and the Taiwan Strait.
