# Supplementary material for: Co-Occurrence Relationship and Stochastic Processes Affect Sedimentary Archaeal and Bacterial Community Assembly in Estuarine–Coastal Margins
Source: Microorganisms. 2022 Jul 1;10(7):1339. doi: 10.3390/microorganisms10071339 (PMC9318014; doi:10.3390/microorganisms10071339)
Supplement: Supplementary file 1 [file microorganisms-10-01339-s001.zip › microorganisms-1797082-SI.pdf]

Supporting Information

Table S1. The environmental variables of sediments at sampling points in Jiulong River Estuary and Taiwan Strait.

Area	Classification	Sample	Depth (m)	Temp (℃)	Sal (‰)	pH	Chla (μg/L)	DO (mg/L)	BGA-PE (μg/L)	TDS (mg/L)	NH_3-N (mg/kg)	TN (%)	TOC (%)
Jiulong River Estuary	L	L1	7.8	18.1	2.36	7.77	3.68	6.07	6.07	2866	120.51	0.15	1.28
		L2	7.6	17.83	4.23	7.75	4.46	6.24	10.78	4954	108.61	0.13	1.16
		L3	11	17.58	6.57	7.64	3.87	6.56	9.6	7460	105.1	0.12	1.1
		L4	9.3	17.36	9.34	7.72	3.15	6.93	9.53	10337	97.45	0.13	1.2
	M	M5	9.8	17.04	13.35	7.75	2.63	7.37	7.67	14366	57.51	0.11	1.17
		M6	6.9	17.06	13.2	7.73	2.54	7.21	7	14215	22.93	0	0.24
		M7	6.9	17.18	13.88	7.79	2.83	7.32	8.3	14891	62.07	0.1	1.13
		M8	5.8	17.31	14.06	7.75	3.24	7.35	7.86	15067	18.54	0.05	0.67
		M9	5.7	17.56	15.13	7.8	3.11	7.73	7.58	16113	34.17	0.05	0.71
		M10	6.6	17.17	17.91	7.98	2.76	7.83	6.41	18797	119.54	0.13	1.21
	H	H11	7.2	17.04	21.05	7.99	3.96	7.99	5.78	21777	10.03	NA	NA
		H12	9.5	16.95	23.17	8.02	1.85	8.11	5.59	23766	18.03	0.06	0.84
		H13	10	16.84	25.66	8.04	1.82	8.1	5.5	26067	52.02	0.1	0.9
		H14	10.5	16.66	26.62	8.05	1.85	8.12	6.08	26947	48.19	0.09	0.89
Taiwan Strait	X	X1	16.5	22.16	30.04	7.97	1.44	6.92	3.96	30054	30.81	0.11	0.87
		X2	19	20.84	31.04	NA	NA	NA	NA	NA	17.71	0.03	0.34
		X3	44	21.25	32.24	NA	NA	NA	NA	NA	16.34	0.03	0.31
	Q	Q1	31.8	21.16	31.26	8.07	1.54	7.41	4.31	30747	57.37	0.12	0.94
		Q2	19	21.21	31.1	8.12	2.13	7.41	5.04	30594	24.68	0.05	0.52
		Q3	31.7	21.74	31.5	8.14	1.62	7.42	4.02	30919	32.91	0.06	0.51
		Q4	57.6	23.17	33.63	8.18	1.39	7.2	3.45	32838	25.88	0.02	0.17
		Q5	47	24.74	34.42	NA	NA	NA	NA	NA	24.27	0.04	0.22
	P	P1	43	20.34	30.88	NA	NA	NA	NA	NA	29.48	0.09	0.73

	P2	51	23.17	33.96	NA	NA	NA	NA	NA	23.2	0.07	0.56
	P3	24	19.96	30.17	NA	NA	NA	NA	NA	16.27	0.12	0.84
	P4	34	20.38	30.68	NA	NA	NA	NA	NA	15.54	0.09	0.68
	P5	76	23.31	34.08	NA	NA	NA	NA	NA	16.89	0.03	0.19
E	E1	48	22.21	32.19	8.15	1.86	7.27	4.89	31527	27.31	0.15	0.86
	E2	65	23.49	33.81	8.17	1.41	6.83	3.69	33009	18.01	0.09	0.61
	E3	21.2	20.49	29.25	8.07	1.19	7.65	3.87	28934	19.51	0.08	0.85
	E4	49	21.71	30.71	8.13	1.71	7.24	4.66	60659	20.15	0.13	0.84
	E5	58	22.18	32.89	NA	NA	NA	NA	NA	19.83	0.13	0.9
	E6	74	23.75	34.12	NA	NA	NA	NA	NA	14.58	0.07	0.5

Table S2. Sequences number and alpha diversity indices of sediments in Jiulong River Estuary and Taiwan Strait.

Classification	Sample name	Archaea				Bacteria			
		Sequences number	OTU	Chao1	Shannon	Sequences number	OTU	Chao1	Shannon
L	L1	24686	322	322	7.86	19829	119	119	4.93
	L2	31484	198	198	7.35	22426	257	257	6.07
	L3	17520	173	173	7.11	13533	99	99	3.71
	L4	27496	208	208	7.4	29600	217	217	4.92
M	M1	19372	96	96	6.08	17723	262	262	7.15
	M2	21758	81	81	5.87	20868	318	318	7.42
	M3	37214	82	82	5.77	19915	204	204	4.67
	M4	22126	50	50	5.15	15725	308	308	7.35
	M5	22577	103	103	6.1	19374	328	328	7.5
	M6	29586	153	153	6.88	15638	218	218	6.94
H	H1	29807	103	103	6.27	8156	230	230	7.09
	H2	15573	87	87	5.81	35844	603	603	8.34
	H3	19222	58	58	5.35	20931	272	272	7.26
	H4	29572	87	87	6.02	34672	390	390	7.83
X	X1	29310	317	317	7.88	19459	232	232	6.8
	X2	26618	452	452	8.14	27391	424	424	6.66
	X3	42398	621	621	8.53	23401	453	453	8.01
Q	Q1	30952	116	116	6.29	15212	110	110	3.75
	Q2	14173	100	100	6.13	17260	187	187	6.79
	Q3	14190	128	128	6.41	15304	200	200	6.77
	Q4	28855	214	214	7.26	22176	319	319	7.68
	Q5	16997	169	169	6.76	22285	117	117	4.22
P	P1	21413	186	186	7.09	19433	330	330	7.56
	P2	18783	298	298	7.66	33695	571	571	8.31

	P3	21316	344	344	8.02	31446	481	481	8.19
	P4	23240	401	401	8.09	22834	262	262	5.5
	P5	28617	239	239	6.63	35419	742	742	8.78
E	E1	22199	70	70	5.6	21118	366	366	7.06
	E2	19203	167	167	6.82	25555	256	256	4.33
	E3	11689	83	83	5.82	17034	254	254	7.32
	E4	18628	146	146	6.64	24013	402	402	7.89
	E5	42799	350	350	7.98	25127	388	388	7.8
	E6	11175	151	151	6.84	29222	843	843	8.85

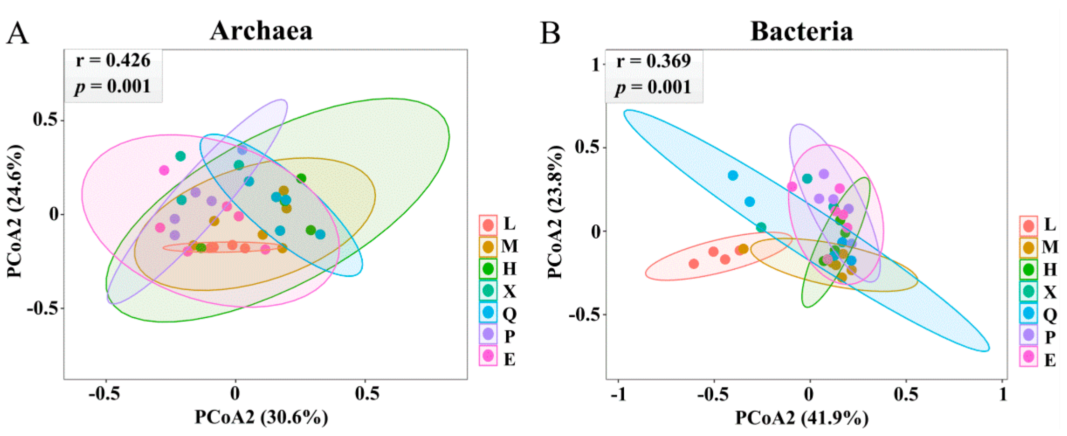

Figure S1. Principal co-ordinate analysis (PCoA) of the archaeal (A) and bacterial (B) communities in Jiulong River Estuary and Taiwan Strait, based on the unweighted UniFrac distance metric. Different circles indicate sediments samples were divided into different cluster patterns.

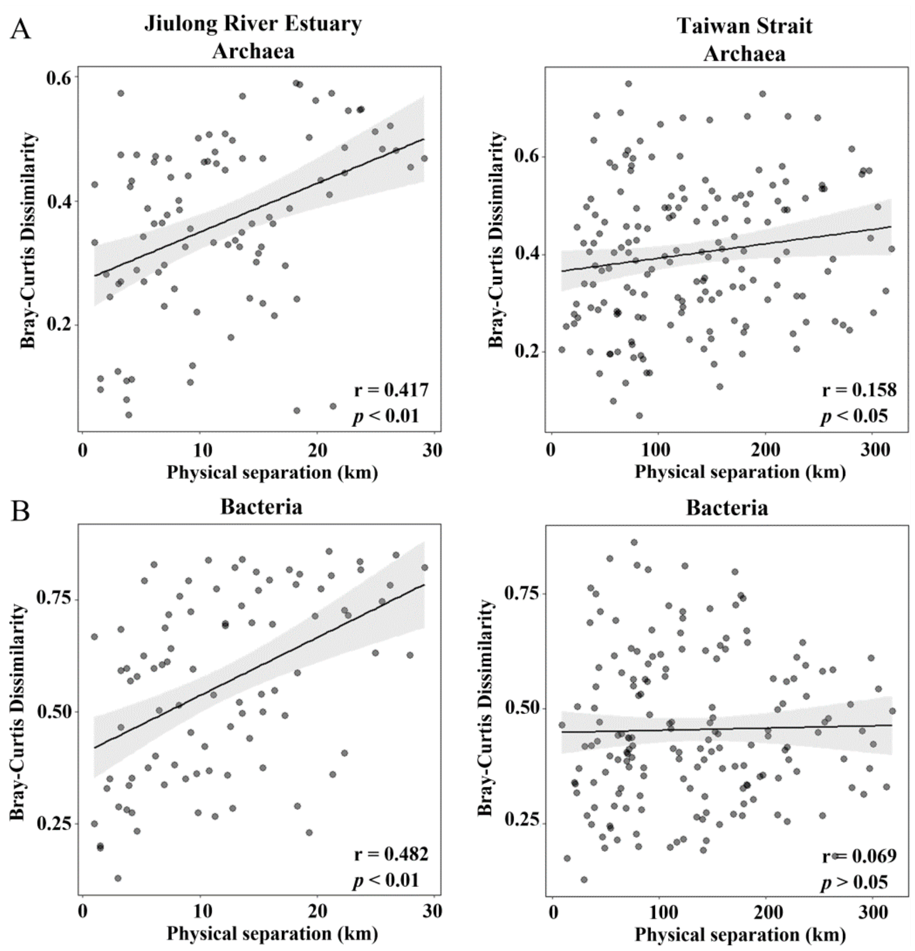

Figure S2. Distance–decay patterns of the archaeal (A) and bacterial (B) community and geographical distance in Jiulong River Estuary and Taiwan Strait, based on Bray–Curtis dissimilarity. The black line indicates the fit between geographical distance and Bray–Curtis dissimilarity.

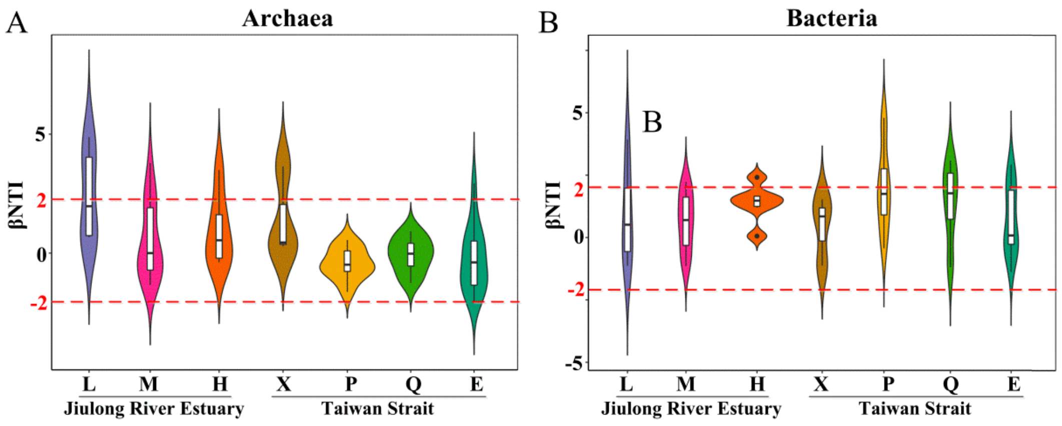

Figure S3. Null model analysis revealing the assembly processes of archaeal (A) and bacterial (B) community in Jiulong River Estuary and Taiwan Strait.
